# Improved self- and external assessment of the clinical abilities of medical students through structured improvement measures in an internal medicine bedside course

**DOI:** 10.3205/zma001058

**Published:** 2016-08-15

**Authors:** S. M. Fünger, H. Lesevic, S. Rosner, I. Ott, P. Berberat, C. Nikendei, C. Sonne

**Affiliations:** 1 Technical University Munich, German Heart Centre, Munich, Germany; 2 Technical University Munich, Klinikum Rechts der Isar, TUM MeDiCAL, Centre of Medical Education, Munich, Germany; 3 Heidelberg University Hospital, Department of General Internal Medicine & Psychosomatic, Heidelberg, Germany; 4 Praxis, Maroussi, Greece

**Keywords:** medical history, physical examination, medical education, e-learning, evaluation, self-assessment, medical examination

## Abstract

**Background: **Bedside courses are of outstanding importance when training medical students. The fact that less and less teaching is taking place nowadays at the patient's bedside makes it all the more important that the available time be put to effective use. The aim of this study was to check whether structured improvement measures in the course (scripts, lecturer briefing, e-learning cases) would improve the abilities of the students on the basis of a subjective self-assessment as well as an external assessment by the lecturers with respect to clinical abilities.

**Methods: **Bedside teaching takes place in the fourth study year in the Medical Clinics of the TU Munich. Both students and lecturers had the chance to hand in an anonymous, quantitative self- and external assessment of the clinical abilities of the students (German grading system) after every course date. This assessment took place online in the three categories "Medical history & examination", "Diagnosis" and "Therapy". An overall period of four semesters, each with 6 course dates, was investigated. After two of the total of four semesters in the study, the course was changed by introducing scripts, lecturer briefing as well as interactive e-learning cases. The self- and external assessment was compared both within the semester (date 1-3: A; date 4-6: B), during the course as well as before and after introducing the improvement measures ("before" (T0): SS 2012, SS 2013, "after" (T1): WS 2013/2014, SS 2014).

**Results: **There was a significant improvement in one's own abilities on the basis of the self-assessment within each semester when comparing the first (A) and the last (B) course dates. Moreover, there was a significant improvement in the performances in all three categories when T0 was compared with T1, from both the point of view of the students ("Medical history & examination": T0 =2.5±0.9, T1=2.2±0.7, pp<0.001; "Diagnosis" T0=3.1±1.0, T1=2.8 ±0.9, pp<0.001; "Therapy": T0=3.8±1.3, T1=3.5±1.2, pp<0.018) and in two of the three categories from the point of view of the lecturers ("Diagnosis": T0=3.0±1.0, T1=2.7±0.7, p.=0.028; "Therapy": T0=3.8±1.1, T1=3.1±1.0, p<0.001).

**Summary: **The structured measures to improve the course including the interactive e-learning cases could have contributed to improved practical abilities with respect to the medical history and examination techniques as well as diagnostic and therapeutic thinking. The external evaluation by lecturers confirmed the improvement with respect to the diagnostic and therapeutic abilities. They only saw no dynamic change in the student's taking histories and clinical examinations.

## Zusammenfassung

**Hintergrund: **Bedside-Kurse sind in der Ausbildung von Medizinstudierenden von herausragender Bedeutung. Die Tatsache, dass heutzutage immer weniger Unterricht am Krankenbett stattfindet, lässt es umso wichtiger erscheinen, die vorhandene Zeit effektiv zu nutzen. Ziel der Studie war es zu überprüfen, ob sich durch strukturierte Verbesserungsmaßnahmen des Kurses (Skripte, Dozentenbriefing, E-Learning-Fälle) die Fähigkeiten der Studenten auf Basis einer subjektiven Selbsteinschätzung sowie einer Fremdbeurteilung durch die Dozenten im Hinblick auf klinische Fähigkeiten verbessern.

**Methoden:** Im vierten Studienjahr findet in den Medizinischen Kliniken der TU München ein Bedside-Teaching statt. Sowohl Studenten als auch Dozenten hatten nach jedem Kurstermin die Möglichkeit eine anonyme, quantitative Selbst- und Fremdeinschätzung der klinischen Fähigkeiten der Studenten abzugeben (deutsches Notensystem). Diese Einschätzung erfolgte online in den drei Kategorien „Anamnese & Untersuchung“, „Diagnose“ und „Therapie“. Insgesamt wurde ein Zeitraum von vier Semestern mit je 6 Kursterminen untersucht. Nach zwei der insgesamt vier untersuchten Semester wurde der Kurs durch die Einführung von Skripten, einem Dozentenbriefing sowie interaktiven E-Learning Fällen verändert. Die Selbst- und Fremdeinschätzung wurde sowohl innerhalb der Semester (Termin 1-3: A; Termin 4-6: B), im Laufe des Kurses, als auch vor und nach Einführung der Verbesserungsmaßnahmen („vorher“ (T0): SS 2012, SS 2013, „nachher“ (T1): WS 2013/2014, SS 2014) verglichen.

**Ergebnisse: **Innerhalb eines jeden Semesters zeigte sich auf Basis der Selbsteinschätzung eine signifikante Verbesserung der eigenen Fähigkeiten beim Vergleich der ersten (A) mit den letzten (B) Kursterminen. Außerdem zeigte sich eine signifikante Verbesserung der Leistungen in allen drei Kategorien beim Vergleich von T0 zu T1 sowohl aus der Perspektive der Studenten („Anamnese & Untersuchung“: T0 =2,5±0,9, T1=2,2±0,7, p<0,001; „Diagnose“: T0=3,1±1,0, T1=2,8 ±0,9, p<0,001; „Therapie“: T0=3,8±1,3, T1=3,5±1,2, p<0,018) sowie bei zwei der drei Kategorien aus der Sicht der Dozenten („Diagnose“: T0=3,0±1,0, T1=2,7±0,7, p=0,028; „Therapie“: T0=3,8±1,1, T1=3,1±1,0, p<0,001).

**Zusammenfassung: **Die strukturierten Maßnahmen zur Verbesserung des Kurses inklusive der interaktiven E-Learning Fälle könnten zu verbesserten praktischen Fähigkeiten im Hinblick auf Anamnese- und Untersuchungstechniken sowie diagnostisches und therapeutisches Denken beigetragen haben. In der Fremdevaluation durch Dozenten konnte die Verbesserung im Hinblick auf diagnostische und therapeutische Fähigkeiten bestätigt werden. Lediglich in der Anamneseerhebung und klinischen Untersuchung der Studenten sahen sie keine Dynamik.

## Notes

The authors Lesevic and Nikendei = shared initial authorship and shared final authorship.

Author Sonne: Master's thesis for the Master of Medical Education, Ruprecht-Karls-University Heidelberg University Hospital 

Masculine names used grammatically in this article apply equally for both male and female persons.

## Introduction

Teaching at the patient's bedside, so-called bedside teaching (BST), has been an important part of training medical students since ancient times [1]. The major part of a human medicine degree course nowadays is made up of lectures and seminars at many places. This teaching is being increasingly supplemented by practical exercises (skill courses, simulation training). The share of teaching that takes place directly at the patient's bedside and that focuses on the patient continues to decrease [2], [3], [4]. Nevertheless, bedside courses are eminently important to learn about the clinical pictures, to practice history-taking and examination techniques, a structured clinical way of thinking, though also to preserve basic "soft skills" [3], [5], [6]. What's more, up to 70% of the correct suspected diagnoses can made by history-taking and physical examinations [7]. The students also expressed a clear wish for more practical exercises [8], [9] and supportive medical assistance when learning the most important medical abilities [10]. Apart from learning clinical abilities, BST offers some decisive advantages. For example, it could be shown amongst other things that bedside courses (BSC) have a positive effect on the doctor-patient relations and promote the patient's understanding of their illness, and thus have significant effects on the attitude of the prospective doctors [3]. Despite the numerous advantages of bedside teaching, its integration in everyday hospital routines often poses problems [3]. Staff shortages as well as tightly scheduled procedures on the wards make a student-based learning environment a real challenge [11], [12]. This make a constant analysis of and improvement in the quality of BST based on feedback from both students and lecturers all the more important. A study by Gonzalo et al. has already described various strategies on the preparation and performance of bedside courses by experienced BSC lecturers, such as a good choice and preparation of patients, explicit assignment of the students' roles, practical instruction of the students on patients and the encouragement of feedback [13]. In our own study, it could be shown that following the introduction of simple, structured improvement measures, the students rated their clinical abilities much higher than beforehand. The measures included materials for a better preparation of the students such as examination scripts, lecturer briefings and the definition of global learning objectives by the lecturers [14]. 

In the fourth study year of the human medicine degree course at the Technical University Munich there is an extensive BSC in internal medicine. The goal of this course is the further development of the history-taking and physical examination techniques that have already been learnt in the third semester. Moreover, the student's diagnostic and therapeutic way of thinking should be improved so that the students are able to draw up a "diagnosis" and therapy plan for the frequent clinical pictures that are presented on their own. Based on the feedback from the online questionnaire, suggestions for concrete improvements to the BST were drawn up and implemented. These include the introduction of scripts, lecturer briefing as well as the possibility of preparation for and follow-up to the course with the aid of e-learning cases. The latter has already been described as an effective method to supplement the direct teaching [15], [16], [17], [18], [19]. 

The aim of the study was to investigate whether and to what extent the introduction of improvement measures enhances the clinical abilities of the students on the basis of the self-assessment as well as an external assessment by the lecturers. In addition to the quantitative parameters, the qualitative free text answers of the students can indicate desired improvements and express their satisfaction with respect to the performance of the course. 

## Methods

### Bedside teaching

The course was held for students in their fourth study year at the Technical University Munich once a week for three hours during the semester (a total of 6 dates per semester). The students visited internal medicine wards of the nephrology, pneumology, cardiology, rheumatology and gastroenterology specialties in small groups of up to eight students. The hospitals involved were the I and II Medical Clinics of the Klinikum Rechts der Isar, the German Heart Centre Munich, the Klinikum Bogenhausen as well as the Barmherzige Brüder Hospital in Munich. 

#### Sample group

The students were supervised by one lecturer on each ward during the course. In the summer semester 2012 (SS 2012) there were 165, in the summer semester 2013 (SS 13) 200, in the winter semester 2013/2014 (WS 13/14) 205 and in the summer semester 2014 (SS 14) 152 students in the course. 60 lecturers were assigned to the course each semester. 

#### Evaluation

All of the students and lecturers were contacted by e-mail after every day of the course and asked to complete an online questionnaire. The evaluation took place anonymously by means of an online questionnaire after every day of the course. This procedure has already been used successfully in previous publications [14], [20]. Both the students and lecturers were reminded about handing in the assessment each week. The evaluation took place over four semesters (SS 12, SS 13, WS 13/14, SS 14). The students were asked for a self-assessment of their performance with respect to the history-taking and examination as well as diagnostic testing and therapy planning as follows:

1. Do you now feel able to perform history-taking and a physical examination?
2. Do you now feel able to draw up a plan for the relevant diagnostic steps for the most common illnesses in internal medicine?
3. Do you now feel able to draw up a plan for the relevant therapeutic steps for the most common illnesses in internal medicine?

The lecturers were asked to assess the students' performance in the same categories:

1. Are the students now able to perform history-taking and a physical examination?
2. Are the students now able to draw up a plan for the relevant diagnostic steps for the most common illnesses in internal medicine?
3. Are the students now able to draw up a plan for the relevant therapeutic steps for the most common illnesses in internal medicine?

The evaluation was carried out using the German school grading system. "1" indicated the best, "6" the poorest grade (1=very good, 2=good, 3=satisfactory, 4=adequate, 5=poor, 6=inadequate). The respondents were also able to make suggestions for improvements ("In general, what would you change?") and say which parts of the course they though were particularly good ("What did you think was particularly good?") in a free text field. 

#### Feedback from the evaluations by students and lecturers

The number of feedbacks from the evaluations by students and lecturers are shown in Table 1 (Tab. 1) and Table 2 (Tab. 2).

#### Structured improvement of the course content and forms of teaching

**Figure 1:**
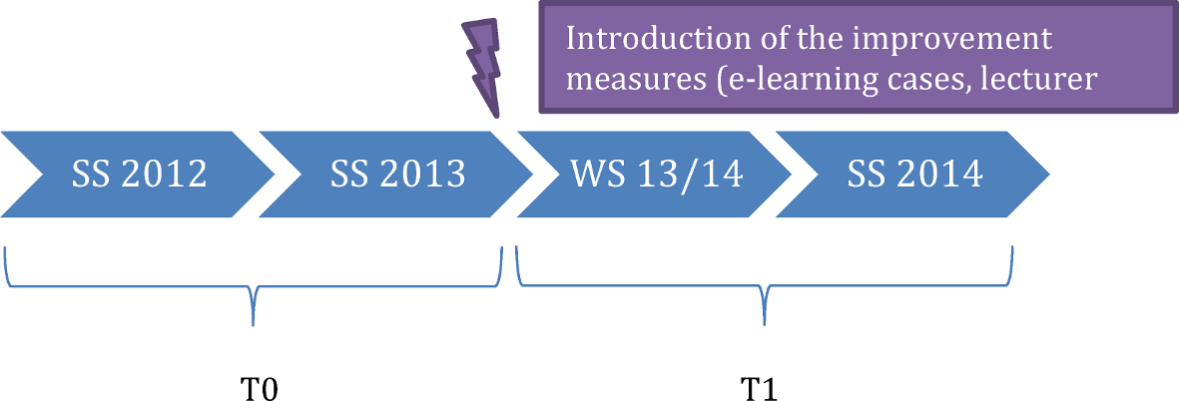
The course design

**Figure 2:**
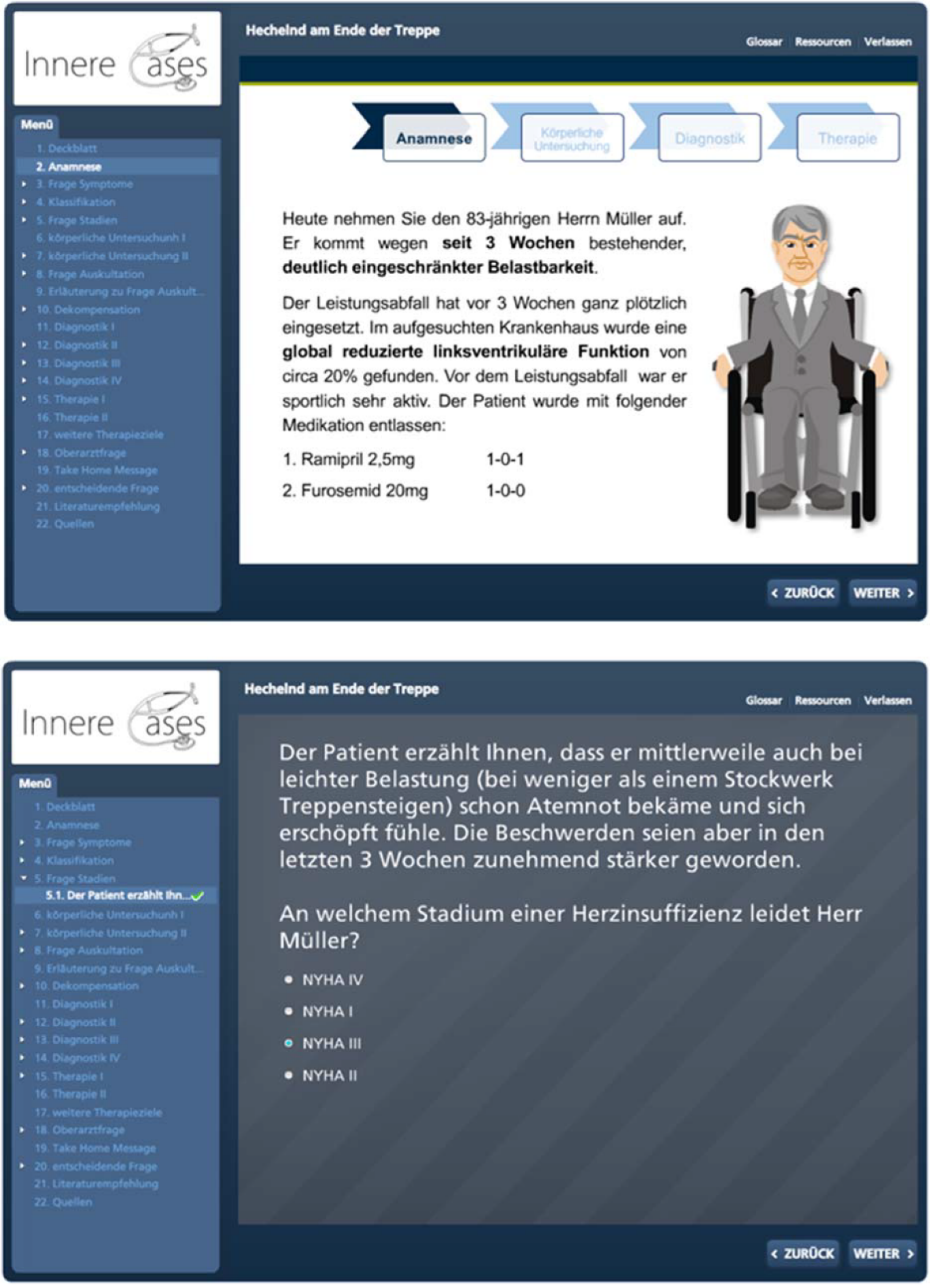
Patients and their symptoms were introduced in the interactive cases.

**Figure 3:**
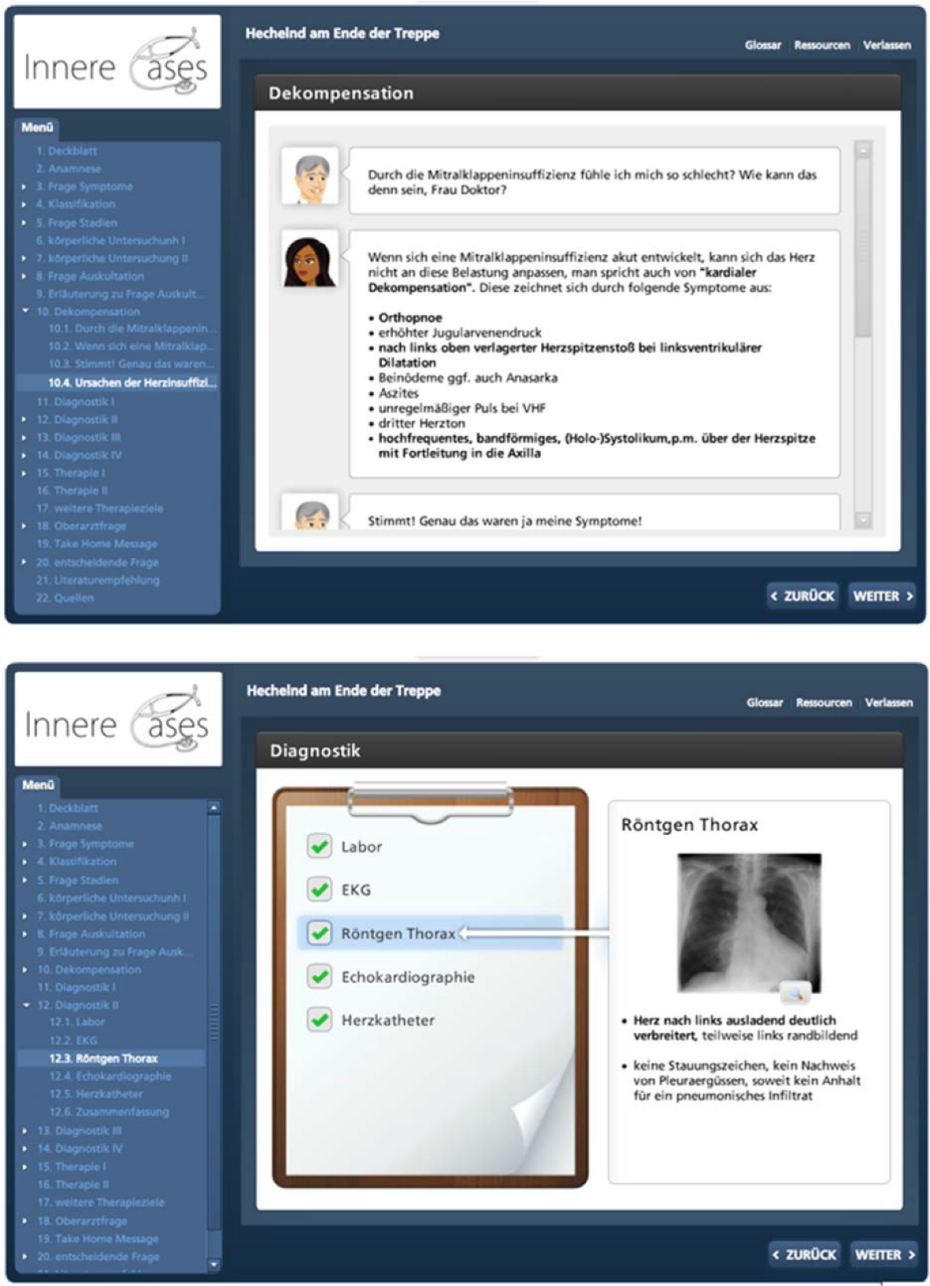
After identifying the symptoms, the illnesses and their characteristic features are dealt with interactively and questions on these are answered.

So as to achieve an improvement in the BSC, the course content was revised and global as well as specific learning objectives were defined. In order to cater for the wishes and suggestions for improvements of the students and lecturers involved, resulting from the evaluations of this course as well as the feedback from students in other studies on the optimisation of practical courses [14], structured improvement measures were integrated in the course structure (see Figure 1 (Fig. 1)). This led to the introduction of scripts in WS 13/14. The scripts contained medical history sheets as well as instructions on how to carry out patient interviews. Structured instructions and tips were also provided on different internal medicine examinations, e.g. a thorax examination. Apart from the physiological findings, pathologies were also often explained along with their clinical evaluation. A targeted preparation for and follow-up to the course by the students was possible thanks to the prior division over the various internal medicine wards and the associated specialist fields. The lecturers were also able to prepare for the individual days of the course with the help of the scripts. In addition to these scripts, a lecturer briefing was held in the morning meetings in each of the hospitals during which all of the lecturers were instructed in the content of the course on the basis of the scripts and the desired evaluation by the students, and during which the importance of supervising the students during history-taking and the physical examination was stressed. Furthermore, a contact person was appointed for the courses whose work included regular reminders of the course and who also helped supervise these. E-learning cases were introduced simultaneously in the winter semester 2013/14. The lecturers were instructed to recommend these to the students as a voluntary form of preparation for and follow-up to the course. These were patient cases that had been created with the program Articulate®, an e-learning software. These cases were taken from the internal medicine disciplines of cardiology, pneumology, gastroenterology, haematology, oncology as well as nephrology and endocrinology. These could be accessed via the Moodle platform of the Technical University Munich, which is open to all students of the TU, after they had registered for the course. The 14 cases constituted an interactive elaboration of a clinical picture on the basis of a concrete patient with his medical history. A quiz in the form of multiple choice questions was linked to information about the respective illness and the associated diagnostic testing and therapy (see Figure 2 (Fig. 2) and Figure 3 (Fig. 3)).

#### Statistical analysis

The grades (man values, MV, and standard deviations, SD) of the first half (date 1-3, A) of the evaluations via the self-/external assessment were tested against those of the second half (B) of the evaluations in each case within each semester. This should allow an assessment of the students' performance in the course of each semester. Two semesters were summarised in each case for an analysis of the development of the students' performance compared to before ("before" (T0): summer semester 2012 and 2013) and after the introduction of the improvement measures ("after" (T1): winter semester 2013/2014 and summer semester 2014). These analyses were also carried out separately for the students and lecturers. Since the questionnaires were completely anonymised, the results could not be evaluated jointly. An explorative statistical analysis of the evaluation share was therefore performed with a Mann-Whitney-U-Test for independent random samples. A p-value of p<0.05 is rated as being statistically significant (SPSS 23, SPSS Inc. Chicago, IL, USA). 

#### Free text answers

The students also had the possibility of providing feedback in the form of a free text answer to the questions "In general, what would you change?" and "What did you think was particularly good?". All free text answers to a question were screened and the tips, suggestions and comments that were provided were then classified in categories. The individual free text answers of the students and lecturers were then assigned to these categories separately for the periods before (T0) and after the introduction of the improvement measures (T1) and their frequency evaluated. The three most common answers in a period were worked out, provided these had been given by at least 10% of the respondents. 

## Results

### Comparison within each semester (A and B)

**Figure 4:**
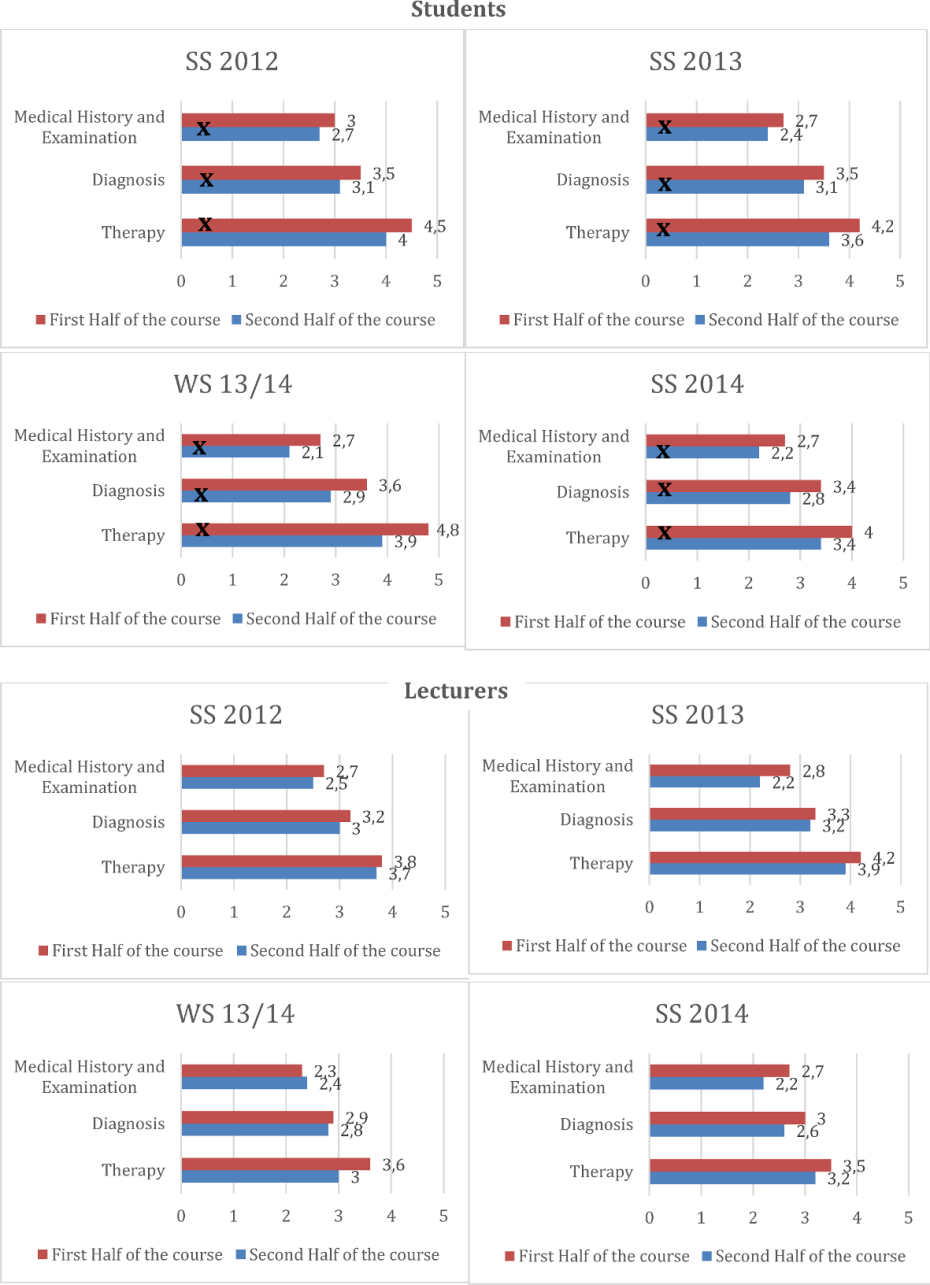
Self-assessment and external assessment by students and lecturers in all four semesters. The results masked with X are significant.

The MV (±SD) of the self-assessments with respect to the categories "Medical history and examination", "Diagnosis" and "Therapy" of the four semesters are shown in Figure 4. The performances of the students improve significantly in the summer semester 2012 according to their self-assessment in the second half of the course (B) in the categories "Medical history & examination" (MV A=3.0±1.1, MV B=2.7±1.0, p.=0.005), and "Diagnosis” (MV A=3.5±1.2, MV B=3.1±1.1, p.=0.004) and "Therapy" (MV A=4.5±1.1, MV B=4.0±1.4, p<0.001) to the performances in the first half of the course (A). A significant improvement can also be identified with respect to the students' self-assessment in the summer semester 2013 ("Medical history & examination": MV A=2.7±0.8, MV B=2.4±0.7, p<0.001, "Diagnosis": A=30.5±0.9, MV B=3.1±0.9, p<0.001, "Therapy": MV A=4.2±1.1, MV B=3.6±1.1, p<0.001). In WS 13/14 the assessments also improve between the first and second half of the course for all three categories ("Medical history & examination“: MV A= 2.7±1.0, MV B=2.1±0.5, p<0.001; "Diagnosis" MV A=3,6±1.1, MV B=2.9±0.9, <0.001; "Therapy": MV A=4.8±1.1, MV B=3.9±1.1, <0.001). A similarly significant effect can be seen for the summer semester 2014 in the three categories ("Medical history & examination“: MV A=2,0.7±0.9, MV B=2.2±0.7, <0.001; "Diagnosis" MV A=3.4±1.0, MV B=2.8±0.9, p<0.001; "Therapy": MV A=4,0.0±1.2, MV B=3.4±1.3, <0.001). The external assessments of the students' performance by the lecturers are also shown in Figure 4 (Fig. 4). The external assessment of the students' performance by the lecturers also improves visually over the semester (see Figure 4 (Fig. 4)). This altered evaluation is not significant. 

#### Comparison before and after the improvement measures (T0 and T1)

**Figure 5:**
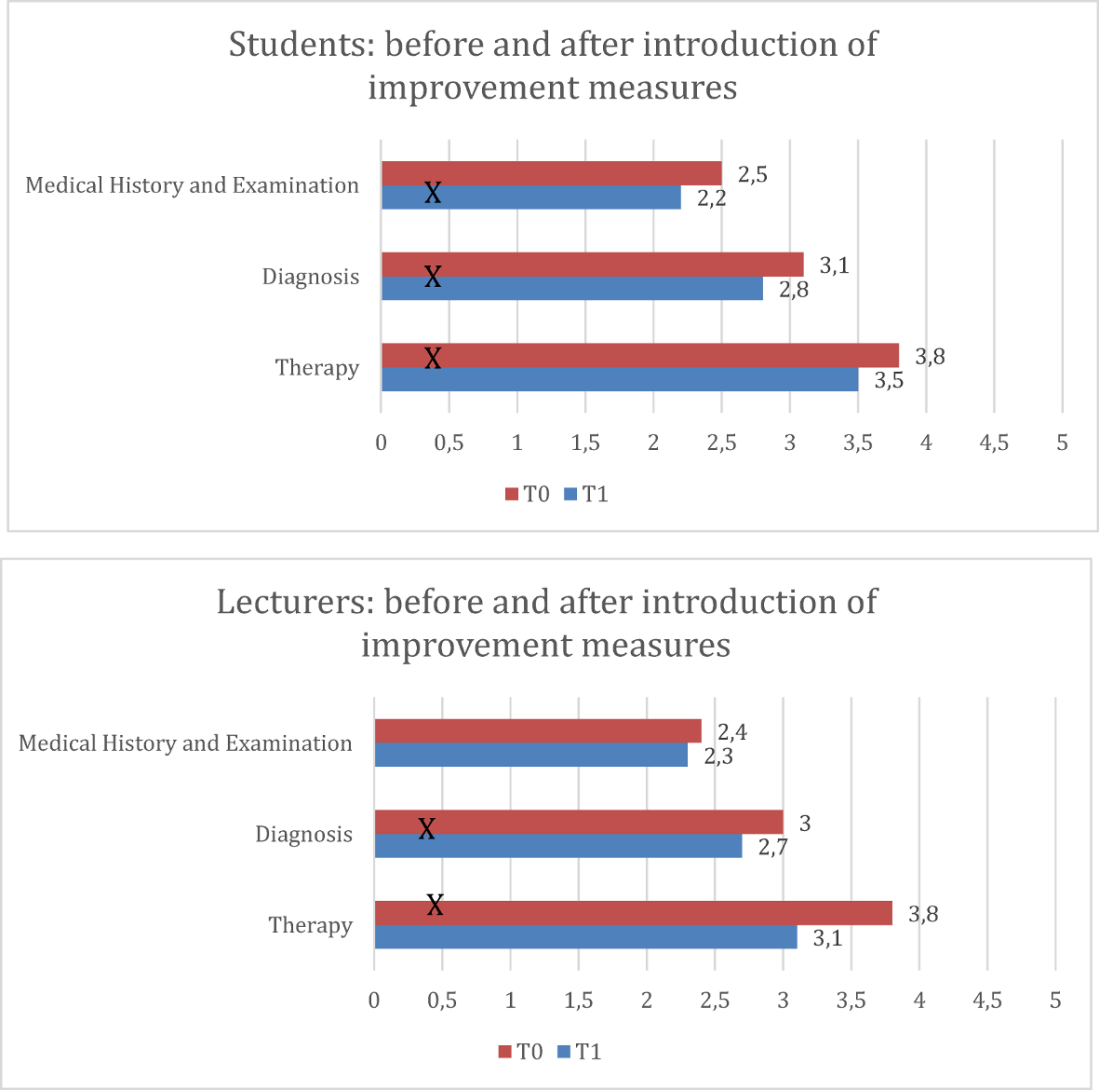
Self-assessment and external assessment by students and lecturers compared before (T0) and after introduction of improvement measures (T1). The results masked with X are significant.

The trend of the students' evaluation as a comparison of T0 and T1 is shown in Figure 5 (Fig. 5). An improvement in all three categories can be seen in SS12 to SS14 in the self-assessment. The difference in the self-assessment grades between T0 and T1 is significant for the categories "Medical history and examination" (MV T0=2.5±0.9, MV T1=2.2±0.7, p<0.001) as well as "Diagnosis" (MV T0=3.1±1.0, MV T1=2.8 ±0.9, p<0.001) and "Therapy" (MV T0=3.8±1.3, MV T1=3.5±1.2, p.=0.018). The trend for the evaluations the lecturers over the years is also shown in Figure 5 (Fig. 5). The students' improvements in the assessment of the lecturers is also visible. The difference between SS 12 and SS 13 on the one hand and between WS 13/14 and SS 14 on the other is significant for the category "Diagnosis" (MV T0=3.0±1.0, MV T1=2.7±0.7, p.=0.038) and "Therapy" (MV T0=3.8±1.1, MV T1=3.1±1.0, p<0.001). The difference in the "Medical history & examination“ is not significant (MV T0=2.4±0.8, MV T1=2.3±0.8, p.=0.798).

#### The free text answers of the students and lecturers 

The free text answers of the students and lecturers for the periods before (T0) and after introduction of the improvement measures (T1) are summarised in table 3 (Tab. 3). 

## Discussion

Bedside courses are essential for teaching fundamental medical skills, such as examination and structured clinical assessment of a patient. We investigated the effect of introducing structured improvement measures with respect to self- and external assessment, with respect to medical history, examination techniques, diagnostic testing and therapy. The study was based on the bedside courses in Munich Technical University. The study groups were also asked about suggested improvements and aspects of the courses which they had particularly liked. It was found that the students considered that the students considered that their performance had significantly improved during a bedside course. This self-observed learning effect was less marked from the perspective of the lecturers. The improvements in the students' self-assessment of their clinical abilities was only found during individual semesters. Moreover, from summer semester 2012 to summer semester 2014, the students' clinical abilities at the patients' bed significantly improved after the improvement measures had been introduced - from the points of view of both the students and lecturers. Aside from the results of the quantitative analysis, the students and lecturers praised and criticized largely similar points - both before and after the introduction of improvement measures. Thus, the students stated that the groups were too large and that they wished more support and feedback from the doctors for the physical examination. However, this was stated by many fewer students after the improvement measures had been introduced. Moreover, many more students praised lecturers' motivation and commitment and the good case discussion after the introduction of E-learning and scripts. Both before and afterwards, the lecturers praised the students' motivation, but criticised their poor prior knowledge and the rooms. 

The results of the qualitative and quantitative analysis were presumably influenced by the systematic and structured improvement in the bedside course by the introduction of scripts, a lecturer briefing and the preparation of the students with E-learning cases. Another factor might be the lecturers' growing experience from the semesters, as these also use the scripts for their preparation of their own students. In our 2013 study, we showed that the self-assessment of the students in practical course significantly improved with the introduction of structured learning goals, examination course scripts and the provision of online material [11]. The results of this study are also consistent with an American study. This study showed that medical students' ability to perform physical examinations was significantly improved by introducing structured instructions, in comparison with unstructured learning [21]. Structured teaching sessions, e.g. in accordance with the sandwich principle, enhance the students' attentiveness and concentration, and thus support more efficient learning processes than frontal mediation, and contribute to the students' independence and a positive learning climate [22]. Moreover, the possible improvement in the students' self-assessment may be linked to their motivation. Previous studies have shown that blended learning – which describes the integration of E-learning and direct teaching - is accompanied in the students' self-assessment by significantly greater motivation, satisfaction and increase in knowledge, in comparison with a conventional course without E-learning [23]. In addition, in a randomised study in students, mediation of medical knowledge by E-learning led to significantly better test results than with conventional lectures [18]. However, the possibility should also be considered that the self-assessed clinical abilities may also have been influenced by parallel curricular elements, such as lectures in internal medicine in the fourth study year, as well as the special subjects and optional courses. 

In the present study, self- and external assessment were recorded with an anonymous online questionnaire. Data collection by self- and external assessment is a recognised approach and has already been used in previous studies [14], [20]. Nevertheless, this procedure has the disadvantage that the subject has to assess his own performance. For this reason, lecturers and students were instructed to use the school marking system in the evaluation, in order to reduce differences in the evaluation criteria for abilities and performance to as great an extent as possible. Moreover, the students' self-assessment could be influenced by the introduction of blended learning [23]. In addition, the present study showed that self-assessment for often more marked for students with poorer performance than for students with better performance [24]. The same study also showed that there was only poor agreement between the students' self-assessment and that of their lecturers. Both these and other studies show that there is low agreement between the students' self-evaluation and external controls of performance [24], [25]. 

Heidelberg University performed a case control study to optimise control. This used BST with the existing curriculum with a structured course containing three different learning units, such as communication training, examination courses and bedside teaching. The number of learning units was the same in both groups. There was no difference between the two groups in a multiple choice test, but there was a significant difference between the two groups in the results of an “objective structured clinical examination” (OSCE) [26]. OSCEs may possibly be a more valid form of checking practical clinical abilities [14]. This test format is regularly used in university medical course [27] and consists of an obstacle course, in which different practical abilities – including taking histories and physical examinations - are checked and examined. 

## Limitations

After the students had been rotated between the individual courses and were therefore taught by different lecturers, the lecturers can only assess the students' momentary status of knowledge and ability, but not their actual individual development or changes in this. Together with the lack of an objective measure of performance, e.g. in the form of an OSCE, can make it more difficult to evaluate the students throughout the course or semester. Moreover, most of the students and lecturers did not report back, so that a representative evaluation is difficult. Because of the anonymous character of the questionnaire, it is impossible to make out whether predominantly high performing or weak students report back. Moreover, there are differences between the semesters with respect to the numbers of reports back for self- and external assessment. While in the winter semester 13/14, 9% of students reported back, the corresponding figure in the summer semester 12 was 35% for the quantitative questions. For lecturers too, the assessments varied from 25% in the summer semester 13 to 55% in summer semester 12. The differences in the numbers of reports back make it more difficult to compare the semesters directly. Another limiting point is the lack of missing data for winter semester 2012/2013; these could not be integrated in this study for technical reasons and which would have been helpful for a complete comparison between different periods. 

## Summary

After introduction of structured measures, such as lecturer briefing, introduction of scripts and provision of E-learning courses for a bedside course, the students considered that their clinical abilities improved significantly. This assessment was partially confirmed by the lecturers. It cannot be conclusively decided whether the students' improved performance in medical history, diagnostic testing and therapy was only due to improvement measures. 

## Competing interests

The authors declare that they have no competing interests.

## Figures and Tables

**Table 1:**
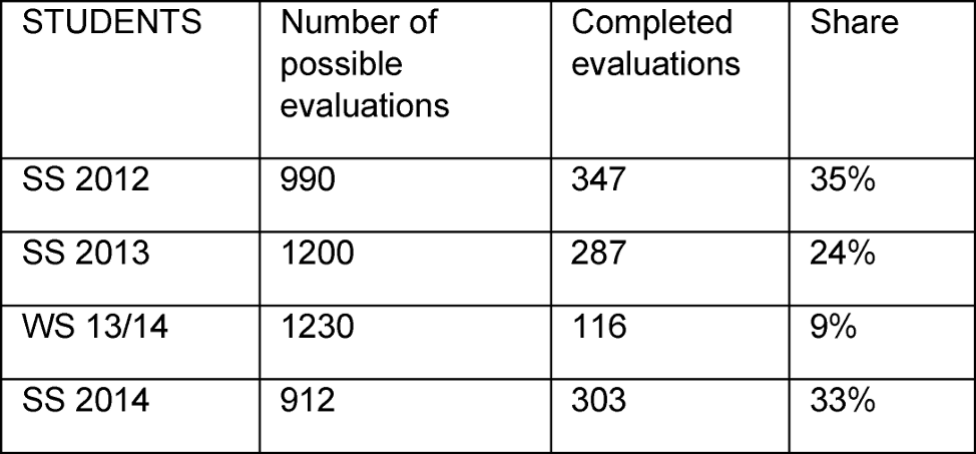
Number of feedbacks from evaluations by students

**Table 2:**
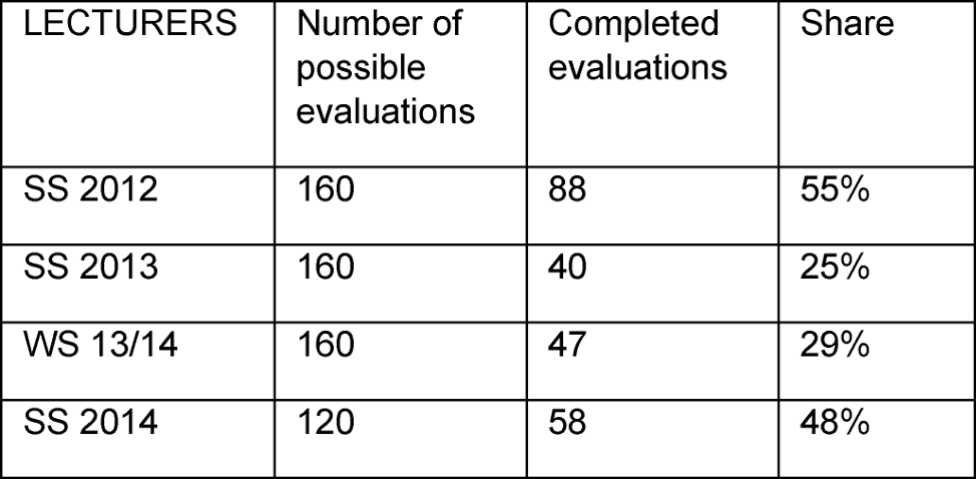
Number of feedbacks from evaluations by lecturers

**Table 3:**
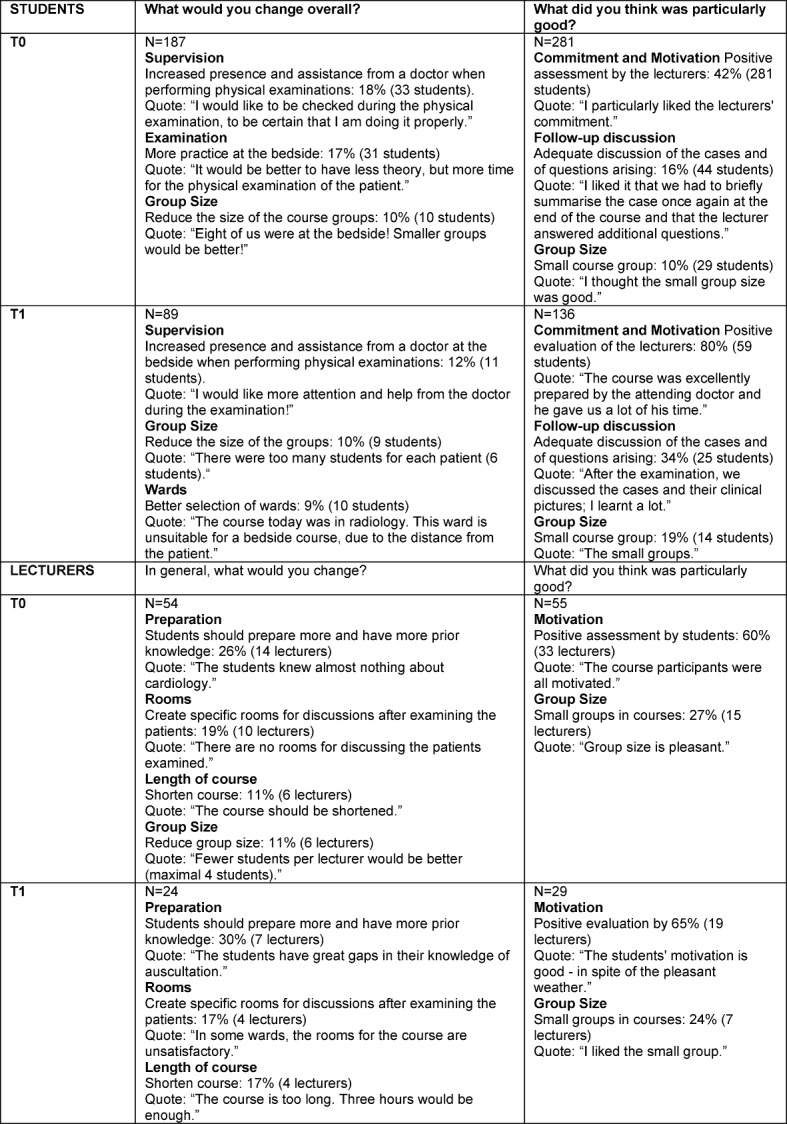
Free text answers from students and lecturers for the periods before (T0) and after introduction of improvement measures (T1)
